# An observational study on the expression levels of MDM2 and MDMX proteins, and associated effects on P53 in a series of human liposarcomas

**DOI:** 10.1186/1472-6890-13-32

**Published:** 2013-12-13

**Authors:** Nader Touqan, Christine P Diggle, Edlo T Verghese, Sarah Perry, Kieran Horgan, William Merchant, Rashida Anwar, Alexander F Markham, Ian M Carr, Rajgopal Achuthan

**Affiliations:** 1 School of Medicine, University of Leeds, Wellcome Trust Brenner Building, St James’s University Hospital, Leeds LS9 7TF, UK; 2 Leeds Teaching Hospitals NHS Trust, Leeds LS9 7TF, UK

**Keywords:** Liposarcoma, MDM2, MDMX, P53, Targeted therapy

## Abstract

**Background:**

Inactivation of wild type P53 by its main cellular inhibitors (MDM2 and MDMX) is a well recognised feature of tumour formation in liposarcomas. MDM2 over-expression has been detected in approximately 80% of liposarcomas but only limited information is available about MDMX over-expression. To date, we are not aware of any study that has described the patterns of MDM2 and MDMX co-expression in liposarcomas. Such information has become more pertinent as various novel MDM2 and/or MDMX single and dual affinity antagonist compounds are emerging as an alternative approach for potential targeted therapeutic strategies.

**Methods:**

We analysed a case series of 61 fully characterized liposarcomas of various sub-types by immunohistochemistry, to assess the expression levels of P53, MDM2 and MDMX, simultaneously. *P53* sequencing was performed in all cases that expressed P53 protein in 10% or more of cells to rule out mutation-related over-expression.

**Results:**

50 cases over-expressed MDM2 and 42 of these co-expressed MDMX at varying relative levels. The relative expression levels of the two proteins with respect to each other were subtype-dependent. This apparently affected the detected levels of P53 directly in two distinct patterns. Diminished levels of P53 were observed when MDM2 was significantly higher in relation to MDMX, suggesting a dominant role for MDM2 in the degradation of P53. Higher levels of P53 were noted with increasing MDMX levels suggesting an interaction between MDM2 and MDMX that resulted in a reduced efficiency of MDM2 in degrading P53. Of the 26 cases of liposarcoma with elevated P53 expression, 5 were found to have a somatic mutation in the *P53* gene.

**Conclusions:**

The results suggest that complex dynamic interactions between MDM2 and MDMX proteins may directly affect the cellular levels of P53. This therefore suggests that careful characterization of both these markers will be necessary in tumours when considering *in vivo* evaluation of novel blocker compounds for MDM proteins, as a therapeutic strategy to restore wild type P53 function.

## Background

Soft Tissue Sarcomas (STS) represent a heterogeneous group of mesenchymal tumours from various tissues of origin that display a spectrum of distributions across the age groups. These relatively rare tumours account for 1% of all cancers and have a poor prognosis, due to high recurrence rates and distant metastasis. The overall five year survival of STS is 50%. This has remained unchanged for the past 15 years [1].

Liposarcomas (LS) account for 20% of STS and are the most common type of STS in adult life. They are morphologically classified into five main subgroups: well-differentiated (WDLS); de-differentiated (DDLS); myxoid (MXLS); round cell (RCLS); and pleomorphic (PLLS). Cytogenetically, WDLS and DDLS characteristically show amplification of the *MDM2* gene [2] and MXLS/RCLS usually have a specific chromosomal translocation t(12;16)(q13;p11) [3]. The mainstay of treatment of LS is radical surgical excision with the use of adjuvant radiotherapy for intermediate and high grade tumours. Approximately 25% of liposarcomas present in the retroperitoneum. At this site, efforts to achieve wide clear surgical margins are more challenging, especially posteriorly, due to anatomical constraints. Conventional chemotherapies have an unproven role in the neo-adjuvant setting. They are mainly prescribed for advanced, inoperable and recurrent sarcomas, but with no significant evidence that they provide an improved survival rate [4]. Therefore new effective, systemic, targeted therapies are clearly needed to improve the outcome for these tumours.

P53 is a key regulator of the cell cycle, apoptosis, DNA repair and cellular senescence [2]. Mutations or deletions in *P53* are seen in approximately 50% of all human cancers [5]. However, the incidence of *P53* mutations in STS had been reported to be significantly lower. Previous analyses have estimated that only 17% of liposarcomas have a *P53* mutation [6,7]. This observation emphasises the important role that other mechanisms probably play, which render wild type P53 inactive in the carcinogenic transformation of liposarcomas.

It is known that non-sarcomatous malignancies with wild type P53 usually demonstrate a clinical pattern that is more responsive to chemotherapy and radiotherapy [6]. This response is not seen in liposarcomas due to the lack of targeted therapies against specific pathways of particular significance in STS formation. The best characterized pathway of this type is the interaction of wild type P53 with its main cellular inhibitors, the “murine double minute” 2 (MDM2) and the “murine double minute” X (MDMX) proteins [8].

MDM2 and P53 regulate each other’s functions through an auto-regulatory feedback loop. Upon activation, P53 promotes transcription of the *MDM2* gene and, in turn, the MDM2 protein inhibits P53 activity. This inhibition is achieved mainly through MDM2 acting as a ubiquitin E3 ligase for P53, thus targeting P53 for proteasomal degradation [9]. Although amplification of the *MDM2* gene is seen in nearly 100% of WDLS and DDLS, over-expression of MDM2 protein is only observed in approximately 75% of these subtypes by immunohistochemistry [10]. High levels of *MDM2* mRNA have been reported as a negative prognostic factor in STS, including liposarcomas [11]. It may be of prognostic significance that the phenomenon of MDM2-mediated P53 inactivation has a predilection to occur more often in retroperitoneal liposarcomas, compared to those that arise in the extremities [12].

MDMX (also known as MDM4) is an MDM2 homolog, which was described after MDM2 [13]. The two proteins share striking structural similarities as both are comprised of an *N*-terminal hydrophobic pocket for P53 binding, a central acidic domain, a zinc-chelating structure and a RING (‘really interesting new gene’) domain of a rare C2H2C4 structural type, for potential binding to generate heterodimers [14]. A significant body of evidence suggests that MDMX is, in addition, an independent negative regulator of P53 [15]. However, in contrast to MDM2, MDMX lacks an intrinsic E3 ligase activity [16] due to structural differences in its RING and central acidic domains, compared to MDM2 [17]. This particular feature of MDMX has provoked some controversy about its exact role in the STS transformation process [18].

Some studies have demonstrated that MDMX enhances the effects of MDM2 by inhibiting the latter’s self-ubiquitinylation and therefore increasing its relatively short cellular half-life. As a result, MDM2 is able to achieve increased P53 degradation [19-21]. MDMX forms heterodimers with MDM2, which also stimulates the ability of the latter to degrade P53 [22]. Other studies, however, have suggested that MDMX may stabilise P53 and, in fact, antagonise the MDM2-targeted degradation of P53 [16,23].

The mutual dependence model described by Gu *et al.*, in modified cell lines, suggested that the two proteins rely on one another to sustain a potent P53 inhibition [19]. The exact cellular functions of MDMX were noted to vary between activation and inhibition of MDM2 depending on the former’s relative expression levels in relation to MDM2 [19]. This model provided an explanation for some of the controversies surrounding MDMX functions in cell lines, in a relatively coherent manner. However, it has previously lacked support from careful descriptive studies performed on actual human sarcoma tissue.

*MDMX* gene amplification had been detected in 17% of human LS [24]. Recent studies have also reported *MDMX* co-amplification with *MDM2* in some STS subtypes, particularly in LS [25,26]. In addition, the over-expression of MDM2 and/or MDMX is generally accepted to correlate with retained wild type P53 [27]. However, previous analysis of MDMX over-expression and of its relative co-expression with MDM2 in human liposarcomas is lacking. In this study, we aimed to characterize various subtypes of adult human liposarcomas in relation to their simultaneous expression levels of MDM2, MDMX and P53. Such a characterization has become a pertinent task due to the exponential growth of MDM2/MDMX single and dual affinity blocking compounds that have emerged in recent years as an attractive targeted therapeutic approach [28-34]. This characterization may also provide insights into the cellular function of MDMX in liposarcomas and may guide future functional studies to evaluate the utility of novel, dual MDM2/MDMX blocking compounds in the treatment of STS [35].

## Methods

### Cohort

Ethical approval was obtained from the Regional Ethics Committee (Leeds Central Research Ethics Committee), approval number 10/H1313/34. A cohort of patients with a fully characterized histopathological diagnosis of liposarcoma, who provided a written informed consent, was identified at the Leeds Teaching Hospitals NHS Trust, UK. The total number of cases was 61 with a median age of 64 years. Details of the clinical cohort are summarised in Table 1.

**Table 1:** The clinical cohort

**Category**	**Subcategory**	**Result (n)**
Sex	Female	31
	Male	30
Anatomical location	Trunk	26 (12 retroperitoneal)
	Extremities	35
Histological subtype	WDLS	39
	DDLS	9
	MXLS/RCLS	12
	Other	1 (Inflammatory)

Summary of the analysed clinical cohort.

### Immunohistochemistry

Formalin-fixed, paraffin-embedded (FFPE) tissue blocks were cut with a microtome at 4 μm thickness, to obtain sequential sections. Sections were floated in a water bath at 39–42°C before transfer onto Superfrost Plus slides. Slides were incubated overnight at 37°C. Slides were de-waxed by serial immersion in a xylene-to-ethanol solvent gradient. Antigen retrieval was performed by immersing the slides in a hot bath of 10 mM citrate buffer (pH 6.0) at 95-98°C for 20 minutes. After cooling for 20 minutes at room temperature, the slides were washed for 5 minutes in deionised water and a further 5 minutes in Tris-buffered saline (TBS) (50 mM Tris.HCl, 150 mM NaCl, pH 7.4). Endogenous peroxidase was blocked by 3% (v/v) hydrogen peroxide for 20 minutes. Slides were washed in TBS for 5 minutes, blocked with 20% (v/v) goat serum in TBS for 30 minutes and then washed in TBS. Incubation with monoclonal primary antibodies diluted in 5% (v/v) goat serum in TBS was performed at the concentrations recommended by the manufacturers, as follows. For MDM2 (Santa Cruz Biotechnology Inc., California, USA, catalogue no. sc-965) at a dilution of 1:250 for 90 minutes; for MDMX (Bethyl Laboratories Inc., Montgomery, USA, catalogue no. IHC-00108-1) at 1:250 dilution; and for P53 (Leica Microsystems, Newcastle, UK, catalogue no. NCL-L-p53-DO7) at a 1:600 dilution. Both of the latter were incubated for 18 hours at 5°C.

To detect the primary antibodies, the NovLink Max Polymer Detection System was used (Leica Microsystems, Newcastle, UK) according to the manufacturer’s instructions. The slides were counter-stained with haematoxylin, dehydrated through an ethanol-to-xylene solvent gradient and mounted under glass cover slips.

Negative control slides were analysed with each cycle of immunohistochemistry and included simple lipomas and normal adipose tissue from human breast specimens.

Scoring was performed on an Olympus multi-viewer light microscope (model number BX41) at 40x magnification. This was done simultaneously but independently by a trained researcher and an experienced histopathologist. Both were blinded to the actual histological diagnosis. 100 cells per slide were scored (maximum of 20 cells per high power field). Particular care was taken to mark the sequential slides of each case identically, so as to score corresponding fields for the three different antibodies.

Only nuclear staining was considered positive. Blood cells, inflammatory cells, non-specific cells and capillary endothelium cells were not included in the scoring process. Scoring was stratified for MDM2 and MDMX as (-, + and ++) where <10, 10–40 and >40 of the 100 cells were stained positive, respectively. P53 was considered over-expressed (+) if 10% or more cells had positive nuclear staining. The slides were then proof read and re-scored separately by a specialist consultant histopathologist.

### Polymerase chain reaction for P53

All 26 cases that over-expressed P53 were analysed. DNA extraction from FFPE blocks was performed using QI Amp^®^ DNA FFPE Tissue Kit (Qiagen, Venlo, Netherlands, catalogue no. 56404). The tissue blocks were first cut using a microtome at 7 micron thickness, the first two sections were discarded and the subsequent 3–5 sections were used, following the manufacturer’s instructions accurately. The resulting extract was then quantified for nucleic acid using a NanoDrop™ 1000 Spectrophotometer. PCR for *P53* exon 4 to 9 and flanking intervening sequences was performed using 6 PCR fragments covering these regions. The sequences were aligned to reference sequence NT_010718.16 (Table 2). PCRs were performed in 25 μl total reaction volumes that contained 0.5 μl of each primer at 10 μM; 0.5 μl of 10 mM deoxynucleoside triphosphates (dNTP); 0.3 μl of *Taq* DNA polymerase; 5 μl of 5x Go *Taq* flexi PCR buffer (Promega, Madison, USA, catalogue no. M890); 1.5 μl of 25 mM MgCl_2_ (Promega, Madison, USA, catalogue no. A531); 2.5 μl of genomic DNA (approximately 50 ng) and brought to the total volume by adding sterile water. Double-stranded DNA was denatured by heating to 95°C for 3 minutes, followed by 40 cycles of the following steps: denaturing at 95°C for 30 seconds; cooling to 50-59°C for 30 seconds; and heating to 72°C for 30 seconds. The final cycle was complemented by an extension at 72°C for 2 minutes. A negative control of all reagents excluding genomic DNA was included in all experiments, as was a positive control of previously analysed DNA (from human blood). The PCR products were then purified from unincorporated oligonucleotide primers using GenElute PCR clean up kit (Sigma Aldrich, St Louis, USA, catalogue no. NA1020), with the manufacturer’s instructions followed accurately.

**Table 2:** P53 primers used in the study

**Exon**	**Nucleotide sequence**	**Size**	**Tm°**
P53 Exon 4	Forward: TCCCAAGCAATGGATGATTT	194 bp	63°C
	Reverse: TTCTGGGAAGGGACAGAAGA		
P53 Exon 5٭	Forward: CTCTTCCTGCAGTACTCCCCTGC	211 bp	55°C
	Reverse: GCCCCAGCTGCTCACCATCGCTA		
P53 Exon 6٭	Forward: GATTGCTCTTAGGTCTGGCCCCTC	182 bp	55°C
	Reverse: GGCCACTGACAACCACCCTTAACC		
P53 Exon 7٭	Forward: GCTTGCCACAGGTCTCCCCAAG	192 bp	59°C
	Reverse: AGGCTGGCAAGTGGCTCCTGAC		
P53 Exon 8٭	Forward: TGGTAATCTACTGGGACGGA	134 bp	50°C
	Reverse: GCTTAGTGCTCCCTGGGGGC		
P53 Exon 9٭	Forward: GCCTCTTTCCTAGCACTGCCCAAC	102 bp	50°C
	Reverse: CCCAAGACTTAGTACCTGAAGGGTG		

Tm°, actual annealing temperature used for each primer; ٭primers reproduced from reference [40].

### DNA analysis

DNA analysis was performed using Sanger sequencing to detect somatic mutations in *P53.* BigDye^®^ Terminator v3.1 Cycle Sequencing Kit (Applied Biosystems Inc. [ABI], Warrington, UK) was used where 1 μl of the template PCR product; 4 μl of BigDye™ 3.1 master mixture and 1.6 picomoles of the corresponding primer were mixed and the solution was brought to 10 μl by adding sterile water. Cycle sequencing was then performed with 25 cycles of rapid thermal ramping with the following steps: 96°C for 10 seconds; 50°C for 5 seconds; and 60°C for 4 minutes. The sequencing reactions were visualised on an Applied Biosystems 3130*xL* Genetic Analyzer. PCR reactions and subsequent sequencing were repeated up to three times in failed cases. Identification, verification and annotation of sequence variants were done using GeneScreen software (http://dna.leeds.ac.uk/genescreen) [36].

## Results

### Immunohistochemistry

The 61 LS cases analysed in the cohort were comprised of the following sub-types: 39 WDLS; 9 DDLS; 12 MXLS/RCLS; and 1 case of inflammatory LS.

As shown in Table 3, MDM2 over-expression (+/++) was detected in 82% of cases (n = 50/61). MDMX co-expression (+/++) was seen in 69% of cases (n = 42/61) in varying ratios compared with MDM2. The co-expression pattern was subtype-dependent, where WDLS displayed abundant levels of MDM2 in relation to MDMX, whereas all other subtypes had comparable levels of MDM2 and MDMX expression. No solitary MDMX (without MDM2) over-expression was detected in any of the analysed cases. 43% of cases (n = 26/61) had positive P53 expression (+). Perhaps unexpectedly, most of these cases (n = 23/26) co-expressed both MDM2 and MDMX as well.

**Table 3:** Score summary of the analysed cohort

	**MDMX -**		**MDMX +**		**MDMX ++**		**Total**
	**P53 -**	**P53 +**	**P53 -**	**P53 +**	**P53 -**	**P53 +**	
MDM2 -	9*	2*	0	0	0	0	11
MDM2 +	3	1	4	0	0	2^†^	**10**
MDM2 ++	4^§^	0	12^§^	1^§^	3^†^	20^†^	**40**
Total	**19**		**17**		**25**		**61**

Immunohistochemistry scores for MDM2 and MDMX were stratified as follows: (-) normal expression: when <11% of cells had positive nuclear staining; (+) moderate over-expression: when 11 – 40% of cells had positive nuclear staining; (++) strong over-expression: when >40% of cells had positive nuclear staining. Scores for P53 were: (-) negative expression: when ≤10% of cells had positive nuclear staining; (+) positive expression: when 11% or more of cells had positive nuclear staining.

*MDM-P53 pathway is inactive as negative staining for MDM2 and MDMX; § collaborative MDM2 and MDMX pattern with higher MDM2 expression levels resulting in negative P53; † competitive MDM2 and MDMX pattern where comparable levels of MDM2 and MDMX expression lead to positive P53.

Eleven cases (10 WDLS and 1 MXLS) had apparently normal (low) MDM2 and MDMX expression levels. Two of these cases also demonstrated P53 over-expression, suggesting the presence of a possible *P53* mutation. However, only one of these two cases was found to have a *P53* mutation on subsequent analysis.

The majority of MXLS/RCLS (11/12) had positive MDM2, MDMX and P53 staining. A predilection to over-express MDMX at higher relative levels was noted in all these cases and all also had positive P53 expression.

Eight of the 29 WDLS that over-expressed MDM2 did not co-express MDMX. This feature was not observed in any of the other subtypes. Seven of these cases also demonstrated negative P53 expression. This may indicate sufficient degradation of P53 by MDM2 alone. Moreover, P53 expression in cases that co-expressed MDM2 and MDMX increased with increasing MDMX levels as might have been predicted by the mutual dependence model. The corollary was also observed, with diminished P53 levels when MDMX expression was present but only at low levels.

### *P53* genomic analysis

All P53 positive cases on IHC (+) were subsequently screened for somatic P53 mutations (n = 26). PCR amplification of exon 7 failed in three of these cases. 5 cases (19%) were found to have previously described pathological mutations: 1 missense mutation in exon 4 c.137C > T; p.S46F in a MXLS that over-expressed MDM2 and MDMX; 3 missense mutations in exon 5 namely c.511G > A; p.E171K and c.392A > G; p.N131S in a WDLS case that had normal MDM2 and MDMX expression; and c.550G > A; p.D184N in two cases (MXLS and WDLS) that over-expressed both MDM2 and MDMX. A frame shift mutation was detected in exon 8 K292*FS (c.876DelAG) in an inflammatory WDLS that also over-expressed MDM2 and MDMX.

## Discussion

In agreement with previous reports, MDM2 over-expression was frequently detected across the various subtypes of LS. MDMX co-expression was also a common feature and probably more frequent than previously reported [24]. In contrast to some previous analyses [12], no specific patterns of MDM2, MDMX or P53 expression were noted in LS, in relation to their anatomical distribution (Table 4).

**Table 4:** Summary of MDM2, MDMX, P53 over-expressions in relation to the anatomical location of LS

**LS subtype**	**Number of cases**	**Retroperitoneal**			**Non-retroperitoneal**		
		**MDM2(+/++)**	**MDMX(+/++)**	**P53(+)**	**MDM2(+/++)**	**MDMX(+/++)**	**P53(+)**
WDLS	39	4/4	2/4	0/4	26/35	19/35	9/35
DDLS	9	7/7	7/7	5/7	2/2	2/2	2/2
MXLS/ RCLS	12	1/2	1/2	1/2	8/10	10/10	9/10

No variations were detected in the expression profiles of the analysed proteins in relation to the anatomical location of LS at the time of presentation.

**Figure 1:**
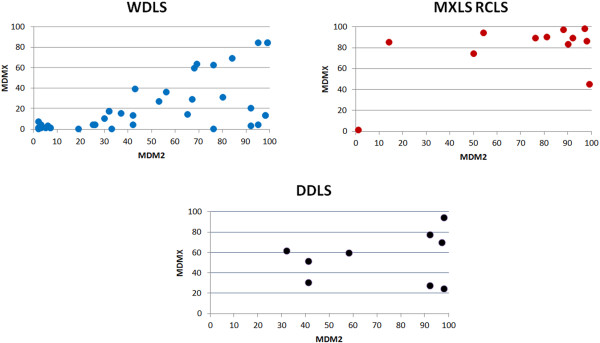
**Co-expression patterns of MDM proteins in different subtypes of liposarcomas.** Different patterns of MDM2/MDMX expression levels were noted across various subtypes of LS. A predilection to over-express MDM2 at higher levels in comparison to MDMX was a feature of WDLS, whereas all other LS subtypes had comparable MDM2 to MDMX expression levels on immunohistochemistry. Data presented are the mean of the individual scores by independent scorers.

The pattern of MDM2 and MDMX co-expression varied between the different subtypes of LS, with a notable tendency for higher expression levels of MDMX in all MXLS and RCLS that over-expressed the two proteins. On the other hand, WDLS predominantly over-expressed MDM2. The distribution of the actual expression values across the different subtypes of LS in the analysed cohort is illustrated in Figure 1.

Most of the cases that did not over-express MDM2 or MDMX had apparently normal expression levels of P53 (n = 9/11). This may indicate that the P53- MDM pathway was intact in these cases. Therefore these biomarkers remained at low levels and, in turn, this might suggest a different mechanism of carcinogenesis in these cases.

**Figure 2:**
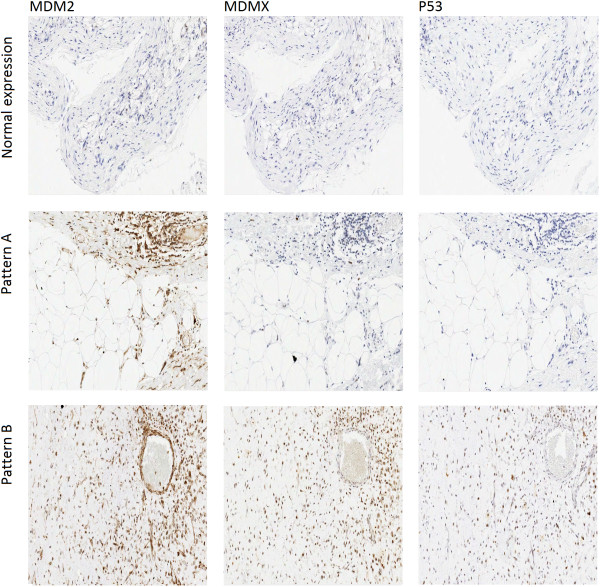
**Immunohistochemistry patterns for MDM2, MDMX and P53.** Three distinct patterns of immunohistochemistry staining were identified: normal expression where none of the examined proteins was over-expressed; negative P53 expression with higher scores of MDM2 in comparison to MDMX (Pattern A); positive P53 expression with comparable scores of MDM2 and MDMX (Pattern B).

MDM2 and MDMX co-expression was noted to influence the expression levels of P53 in two distinct patterns as shown in Figure 2: A) a collaborative pattern where diminished or no P53 expression was detected. This pattern was mainly observed in cases where MDMX was co-expressed with MDM2 but at relatively lower levels (+) (§ in Table 3). This pattern could have resulted from MDMX collaborating with MDM2 to inhibit P53 by targeting it for degradation, leading to diminished P53 expression, in line with MDMX acting as an MDM2 stabiliser. Tumours with this expression pattern may respond well to MDM2 blockers or dual specificity antagonists of higher MDM2 affinity; B) a competitive pattern where higher scores of P53 were observed (>10% of cells). This pattern was mainly noted in cases where MDMX was co-expressed at relatively high levels (++) († in Table 3). This pattern may be explained by MDMX possibly “competing” with MDM2 in binding to P53, resulting in reduced P53 degradation by MDM2 and therefore higher cellular expression levels, in line with MDMX acting, here, as a P53 stabiliser [16,37]. Tumours that express this profile may best be targeted by dual blocker compounds. One could argue that MDMX single affinity blockers may have a reduced therapeutic potential in these cases, as the resulting MDMX-freed P53 may then be subject to degradation by the over-expressed MDM2, if no MDM2 antagonist is used in conjunction. As no cases of LS that over-expressed MDMX exclusively (in the absence of MDM2) were detected, there must be questions about the utility of single affinity MDMX blockers in LS. However, antagonising MDMX-mediated P53 suppression may still have some beneficial therapeutic effect in these cases and functional studies will be needed to clarify this.

**Figure 3:**
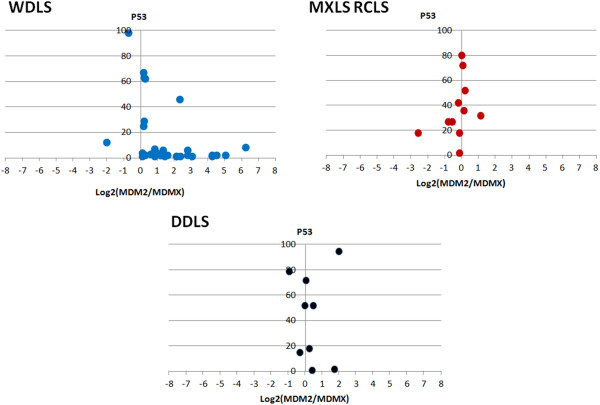
**P53 expressions in relation to the Log2(MDM2/MDMX) scores.** Across various subtypes of LS, higher P53 expression levels were detected when MDMX was co-expressed at comparable or higher levels in relation to MDM2 (Log2(MDM2/MDMX) < 1). Diminished P53 expression was noted when MDM2 was expressed at significantly levels than MDMX (Log2(MDM2/MDMX) > 1). 11 cases that had apparently normal expression of MDM2, MDMX and P53 were excluded from this figure for clarity.

These observations regarding patterns of co-expression are in agreement with the previously described mutual dependence model from modified cell lines. Despite the different genetic alterations involved in the malignant transformation of the different sub-types of LS, it is noted that the expression level of P53 was largely affected by the MDM2/MDMX ratio in all sub-types, with a statistically significant negative correlation between MDM2/MDMX ratio and P53 expression (p < 0.001). P53 expression in relation to the Log2(MDM2/MDMX) scores for each histological subtype is illustrated in Figure 3.

The *P53* genomic analysis data showed a similar mutation rate in cases that overexpressed P53 with those of all LS in general as reported in the literature. 81% of these cases had wild type P53 suggesting that the expression pattern of P53 in these cases is a genuine manifestation of their MDM2-MDMX interactions.

## Conclusion

Our results suggest that the dynamic co-expression of MDM2 and MDMX proteins can directly affect cellular levels of P53. This therefore invites a careful characterization of these markers in tumours when considering *in-vivo* experimental evaluation of novel MDM2-specific or dual target MDM2/MDMX blocking compounds. Ideally, a greater number of samples are required to definitively describe the clinical consequences of the P53-MDM interactions seen in LS tumours. However, due to the rarity of this disease, this study along with other related reports [38,39] can only provide an indication of the importance of the P53-MDM interaction in LS. Nevertheless, given the lack of any current effective targeted therapy for LS patients, we believe further functional studies should be performed to assess the efficacy of this therapeutic approach.

## Abbreviations

DDLS: De-differentiated liposarcoma; FFPE: Formalin-fixed paraffin-embedded; LS: Liposarcoma; MDM: Murine double minute; MXLS: Myxoid liposarcoma; PLLS: Pleomorphic liposarcoma; RCLS: Round cell liposarcoma; STS: Soft tissue sarcoma; WDLS: Well-differentiated liposarcoma.

## Competing interests

The authors declare that they have no competing interests.

## Authors’ contributions

NT carried out the study design, ethical approval, performed all experiments under supervision, data analysis and wrote the manuscript. WM, EV and NT scored all the slides. CD repeated some PCR and sequencing experiments, CD and SP supervised all the lab experiments and reviewed the methodology section. KH and R Achuthan provided the clinical insight for the study and its design. R Anwar and AM made the final corrections of the manuscript. All authors reviewed the final manuscript after corrections.

## Pre-publication history

The pre-publication history for this paper can be accessed here:

http://www.biomedcentral.com/1472-6890/13/32/prepub
